# Evaluation of Water Indices for Surface Water Extraction in a Landsat 8 Scene of Nepal

**DOI:** 10.3390/s18082580

**Published:** 2018-08-07

**Authors:** Tri Dev Acharya, Anoj Subedi, Dong Ha Lee

**Affiliations:** 1 Department of Civil Engineering, Kangwon National University, Chuncheon 24341, Korea; tridevacharya@kangwon.ac.kr; 2 Institute of Forestry, Pokhara Campus, Tribhuvan University, Pokhara 33700, Nepal; anojsubedi99@gmail.com

**Keywords:** water, index method, NDVI, NDWI, MNDWI, AWEI, elevation, Landsat, Nepal

## Abstract

Accurate and frequent updates of surface water have been made possible by remote sensing technology. Index methods are mostly used for surface water estimation which separates the water from the background based on a threshold value. Generally, the threshold is a fixed value, but can be challenging in the case of environmental noise, such as shadow, forest, built-up areas, snow, and clouds. One such challenging scene can be found in Nepal where no such evaluation has been done. Taking that in consideration, this study evaluates the performance of the most widely used water indices: Normalized Difference Vegetation Index (NDVI), Normalized Difference Water Index (NDWI), Modified NDWI (MNDWI), and Automated Water Extraction Index (AWEI) in a Landsat 8 scene of Nepal. The scene, ranging from 60 m to 8848 m, contains various types of water bodies found in Nepal with different forms of environmental noise. The evaluation was conducted based on measures from a confusion matrix derived using validation points. Comparing visually and quantitatively, not a single method was able to extract surface water in the entire scene with better accuracy. Upon selecting optimum thresholds, the overall accuracy (OA) and kappa coefficient (kappa) was improved, but not satisfactory. NDVI and NDWI showed better results for only pure water pixels, whereas MNDWI and AWEI were unable to reject snow cover and shadows. Combining NDVI with NDWI and AWEI with shadow improved the accuracy but inherited the NDWI and AWEI characteristics. Segmenting the test scene with elevations above and below 665 m, and using NDVI and NDWI for detecting water, resulted in an OA of 0.9638 and kappa of 0.8979. The accuracy can be further improved with a smaller interval of categorical characteristics in one or multiple scenes.

## 1. Introduction

Surface water is a vital part of Earth’s ecosystem. It is essential for the survival of living beings [1] and is an excellent indicator of environmental change [2]. Accurate and up-to-date information of the spatial distribution of surface water is a backbone for numerous scientific tasks, such as surface water inventory mapping, water estimation for drinking and irrigation purposes, land use/land cover (LULC) mapping and change, etc. Remote sensing is a rapidly growing technology that can provide low-cost and reliable information for environmental changes at local, regional, and global scales, with their long-collected repeatable, and even real-time, data [3,4]. Numerous water extraction algorithms have been developed and applied for remotely sensed imageries. Statistical pattern recognition techniques, including supervision that uses ground truth data [5,6,7,8], and unsupervised classification methods that first search for endmembers [6,8,9]. Linear unmixing methods use endmembers to unravel the image spectra and, in this case, decide on the fraction of water within each pixel spectrum [10]. The thresholding method uses either a break or range threshold in a single reflectance band [11,12,13] or derived spectral indices [14,15,16,17,18,19] or transformed bands [1] to identify water features. The thresholding method is quite fast and simple to use, which is why they are widely used for identification of water features [20,21,22].

McFeeters developed the most fundamental water index Normalized Difference Water Index (NDWI) [19] using green and near-infrared (NIR) bands of a Landsat Thematic Mapper (TM) image to maximize water feature identification. McFeeters proposed a zero threshold to separate water with background [19], but Xu [17] noted that the zero threshold was not able to separate water bodies over built-up areas. Xu replaced the near infrared (NIR) band in McFeeters’s NDWI with the shortwave infrared (SWIR) band and derived the modified NDWI (MNDWI) [17] to resolve the issue. However, still, there were major problems due to shadows in mountainous terrain. Feyisa et al. [15] proposed the Automated Water Extraction Index (AWEI) to identify water features, which has two conditions: AWEIsh is designed primarily to remove shadow pixels, whereas AWEInsh is designed for areas with an urban background. Furthermore, many other indices have been formulated by various studies for various sensors and areas with varying accuracies [14,23,24,25]. Similarly, vegetation indices have also been used to extract water features [26]. In some cases, the combined approach of different indices and with elevation derivatives has been used to extract water features [27,28]. Lu et al. [29] used the combined difference between Normalized Difference Vegetation Index (NDVI) and NDWI to enhance the contrast between water bodies and the surrounding surface features; the topographic slope to eliminate the mountain shadow; and the NIR band to reduce the effects of artificial construction land. Similarly, Menarguez [30] combined three water indices, namely, Land Surface Water Index (LSWI) [25], MNDWI, and NDWI with the Enhanced Vegetation Index (EVI), and NDVI), and the results revealed that this integrated method was more sensitive to water bodies, especially the mixed water and vegetation pixels. Jiang et al. [31] proposed an automated method for extracting rivers and lakes by combining the water indices (NDWI, mNDWI, AWEIsh, and AWEInsh) with digital image processing techniques in Landsat scenes with mixed water pixels in narrow rivers or shallow water at the edge of lakes or wide rivers in China. A recent review by Huang et al. [32] provides more detailed information on detecting, extracting, and monitoring surface water using optical remote sensing.

Although there are developments in index methods, a major hindrance is the requirement of a threshold that separates water from environmental noises such as shadow, forest, built-up areas, snow, and clouds. These noisy environmental land surfaces mimic water. As the threshold may vary among these varying conditions, most studies are performed on large water bodies with either selected areas or uniform scene conditions [5,26,33,34]. There are very few such studies that have used complex scenes containing more than one water mimicking feature. In such cases, standard threshold values of a single index do not lead to good accuracy and it becomes challenging to identify depending on the area of study, location, weather, and time of acquisition [31]. One such challenging condition for surface water mapping can be found in Nepal. The country is a geographically-diverse country with varying physiology. Once scene can contain elevation ranging from 60 m (Terai) to 8848 m (Himalayas) with surface water of varying temperature, turbidity, depth, and vegetation cover. Even though there are comparison studies of various indices and sensors [33], the evaluation of such in a single scene has never been conducted before, thus, the performance of water indices in the extraction of surface water is unknown.

Hence, in this study, we evaluate the performance of most widely used water indices: NDVI, NDWI, MNDWI, and AWEI in a Landsat 8 scene of Nepal. The scene contains various types of surface water prevalent in Nepal ranging from flat Terai to high Himalayas. The evaluation includes an examination of the normal standard and optimum threshold for surface water. Moreover, the study proposes and evaluates the combination of indices and the segmentation of the test scene with elevations for better surface water detection in the full scene.

## 2. Materials and Methods

The workflow of the study includes: selection of test site, data collection, image pre-processing, derivation of water indices, thresholding, combining, and segmenting the test scene for surface water extraction. Each surface water map will be evaluated qualitatively and quantitatively based on validation points. All of the processing was done in ArcGIS 10.5 (Environmental Systems Research Institute, California, CA, USA), Environment for Visualizing Images (ENVI) version 5.3 (Exelis Visual Information Solutions, Boulder, CO, USA) and R 3.4.4 (The R Foundation, Vienna, Austria) software packages.

### 2.1. Test Area

**Figure 1:**
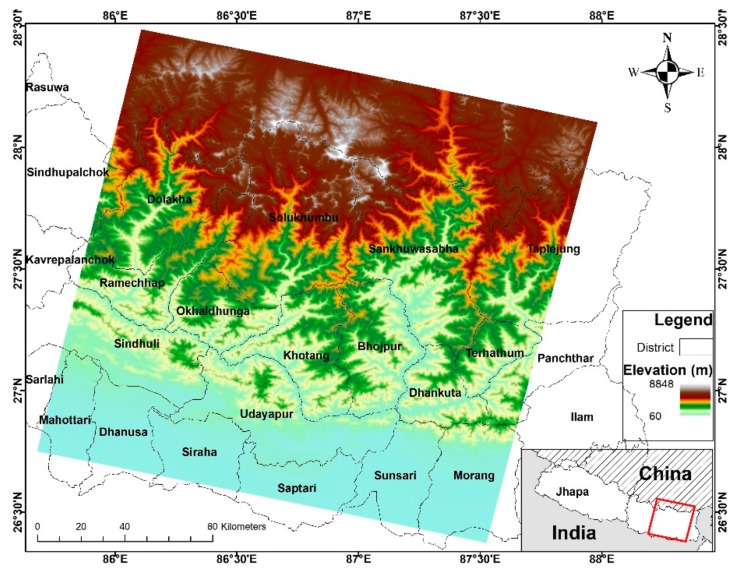
Location map of the test sites in Nepal, along with district boundaries and elevation range.

To evaluate the water indices, a Landsat scene in Eastern Nepal that ranges from Terai to the Himalayas was selected. The test area is 37,127.3 sq. km and situated between 26°21′54.29″ N to 28°28′41.43″ N latitude and 85°41′51.77″ E to 88°0′35.98″ E longitude. Figure 1 shows the location map with elevation and a pansharpened Landsat 8 image of the study area. The area encompasses Bhojpur, Dhankuta, Dhanusa, Dolakha, Ilam, Khotang, Mahottari, Morang, Okhaldhunga, Panchthar, Ramechhap, Sankhuwasabha, Saptari, Sarlahi, Sindhuli, Sindhupalchok, Siraha, Solukhumbu, Sunsari, Taplejung, Terhathum, and Udayapur districts. The elevation ranges from 60 m to 8848 m, containing flat lands in the Southern Terai with deciduous forest, mid-hilly regions with deciduous and coniferous forest, and the remaining rugged mountains with snow, glacial lakes, barren slopes, and few grasslands. The vast range in elevation creates varying conditions of water bodies, thus, this site could be a unique area for evaluation between surface water features and various sorts of environmental noise. The notable areas in the scene are Mount Everest (highest peak), the Koshi River barrage (dammed wide fresh water river), and the Biratnagar Metropolitan area (urban area with ponds).

### 2.2. Data

For this study, a Landsat 8 scene from path 140 and row 41, acquired on 8 December 2017, was collected from the United States Geological Survey (USGS) Global Visualization Viewer (GLOVIS) portal (http://glovis.usgs.gov). The image was Level 1 terrain-corrected (L1T) and pre-georeferenced using the WGS84 datum. The scene centre was 27°25′52.54″ N, 86°49′43.75″ E and had cloud cover of 4.43%. Specifications of the Landsat image are shown in Table 1. The image was pre-processed with the radiometric calibration tool in ENVI which converted the digital number (DN) image to top-of-atmosphere (TOA) reflectance. The tool uses all the required information of the conversion for the Landsat header MTL metadata file.

**Figure 2:**
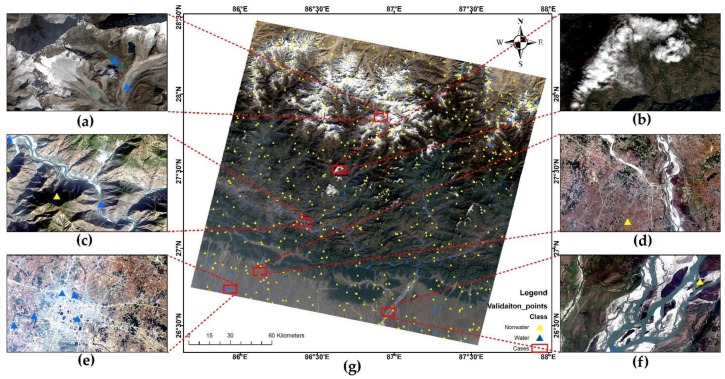
Pansharpened Landsat 8 true-colour composite image with water and non-water reference points (**g**). Each red box represents different case of surface water bodies in the presence of environmental noise (**a**–**f**).

Figure 2g shows the pansharpened Landsat 8 image with validation water and non-water points. Additionally, Figure 2a–f shows different types of water bodies with noisy backgrounds in the scene. Figure 2a shows the cold ice water with snow cover in rugged mountains. Figure 2b shows hilly areas with clouds and shadows. Figure 2c,d show narrow rivers in hills and flat land, respectively. Figure 2e represents urban ponds, whereas Figure 2f represents wide and shallow rivers braided with sand. Using an index and pinpointing the optimum threshold in a scene with such diversity is a challenging task. Hence, we took the scene as our test image in Nepal for the evaluation of water indices for surface water extraction.

To obtain the reference data for accuracy assessment, first, 500 random points were generated over the scene areas and they were labelled as water and non-water with the help of high-resolution images available from Google Earth Pro^TM^ (Google Inc., Menlo Park, CA, USA), pansharpened imagery of Landsat OLI, and field observation. An additional 300 water and noisy non-water points were added based on the field verification and expert’s knowledge of the area for small ponds and narrow rivers and canals. Thus, there is a total of 800 points, of which around 21%, i.e., 186, were water points and the remaining 79% were non-water points.

For the extraction of elevation for the scene, the Shuttle Radar Topography Mission (SRTM) Global Digital Elevation Model (DEM) of 30 m [36] was obtained from the OpenTopography portal (http://opentopo.sdsc.edu). Its version 3.0 product is void-filled and the most complete high-resolution digital topographic database of the Earth. Their evaluation in hilly areas shows a good representation when compared with high-resolution data [37].

### 2.3. Water Indices for Surface Water Extraction

The derivation of water indices and the threshold for binary classification of the water and non-water background is done according to the remarks shown in Table 2.

In the surface water extraction process, first, the threshold of 0 will be used for all of the individual index maps, then an optimum threshold will be searched that will give the highest overall accuracy and kappa coefficient based on the trial and error method. Furthermore, based on the evaluation matrices, indices with better extraction abilities will be combined to increase the separability between water and non-water surfaces. Additionally, as elevation is one of the varying characteristics in the scene, we propose to use elevation for segmentation of the scene and then use a suitable water index for each segment so that it results in a better classification of the full scene.

### 2.4. Accuracy Assessment

Even though the purpose of the indices is to produce fast and accurate water maps, an accuracy assessment must be conducted for evaluation purposes. A confusion matrix-based approach, as shown in Table 3, is used in this study. On comparing the extracted water and non-water map with reference data, the outcomes are four types of pixels:

- True positive (TP): The number of correctly extracted water pixels;
- False negative (FN): The number of undetected water pixels;
- False positive (FP): The number of incorrectly extracted water pixels; and
- True negative (TN): The number of correctly rejected non-water pixels.

Based on above four outcomes, the overall accuracy (OA) and kappa coefficient (kappa) were used to assess the accuracy of the produced maps with different water indices. These can be calculated as:

(1) $$\mathrm{Producer’s accuracy}=\frac{\mathrm{TP}}{\mathrm{TP}+\mathrm{FN}}$$

(2) $$\mathrm{User’s accuracy}=\frac{\mathrm{TP}}{\mathrm{TP}+\mathrm{FP}}$$

(3) $$\mathrm{Overall accuracy}=\frac{\mathrm{TP}+\mathrm{TN}}{T}$$

(4) $$\mathrm{Kappa coefficient}=\frac{T\left(\mathrm{TP}+\mathrm{TN}\right)-\sum }{T^{2}-\sum }$$

where ∑ is the chance accuracy represented by (TP+FP)(TP+FN)+(FN+TN)(FP+TN), and T is the total number of pixels in accuracy assessment.

The PA, UA, and OA represent the correct predictions and ranges from 0 to 1, where a value close to 1 is all perfect. However, it does not consider the agreements between datasets that are due to chance alone. Hence, the kappa, a tool to control for that random agreement factor, is often used together with PA, UA, and OA. Usually, kappa can range from −1 to +1, where 0 represents the amount of agreement that can be expected from random chance, and 1 represents perfect agreement between the raters [39].

## 3. Results and Discussion

In this section, the performance of the various water indices for the surface water extraction for the entire scene is assessed. First, an evaluation of each index will be conducted for the standard 0 and optimum thresholds, then a combination of indices and segmentation with elevation will be explored for possible water extraction. A detailed comparison and discussion of the different water types will be conducted to evaluate the performance by each method.

**Figure 3:**
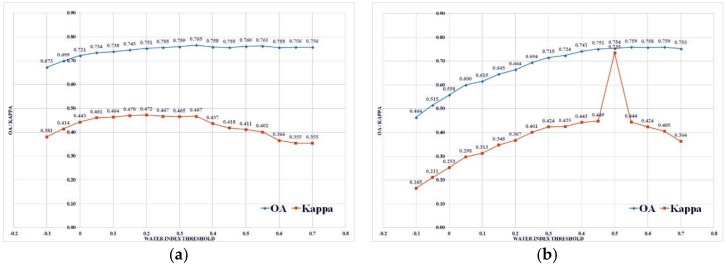
Overall accuracy (OA) and kappa coefficient (kappa) of (**a**) MNDWI1 and (**b**) MNDWI2 using validation points during trial and error.

Using the equations of Table 2, six index maps were derived. For each scene, binary classification was first conducted with the standard 0 threshold. Then, optimum thresholds were selected based on the highest OA and kappa for the validation dataset using the trial and error method (Table 4). For the trial and error method, we first used two-decimal thresholds of a 0.05 interval between −1 and +1 for the validation data to obtain the highest OA and kappa. Once the two-decimal threshold was decided, a smaller interval threshold of 0.0005 was used between ±0.05 of the previously selected two-decimal threshold value. The final four-decimal threshold was chosen for the closest real value in the validation data. For example, 0.4 was selected as the first decimal threshold for NDVI, then the second decimal threshold was tested between 0.35 and 0.45. In the second step, 0.3875 was found to be the four-decimal threshold. As 0.3875 was not in the validation data, the closest 0.3877 was chosen as final optimal threshold for NDVI. Figure 3 shows the example of the plot of the OA and kappa for MNDWI1 and MNDWI2 for an interval of 0.005.

**Figure 4:**
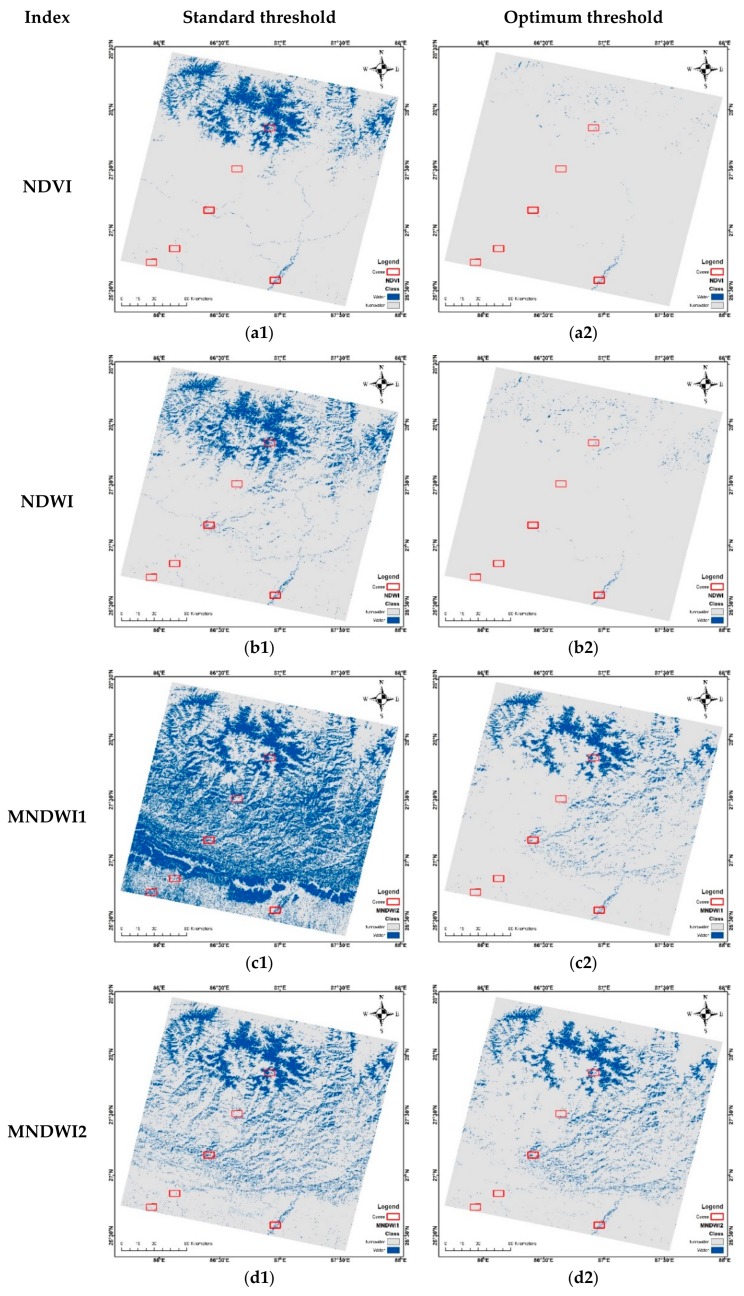

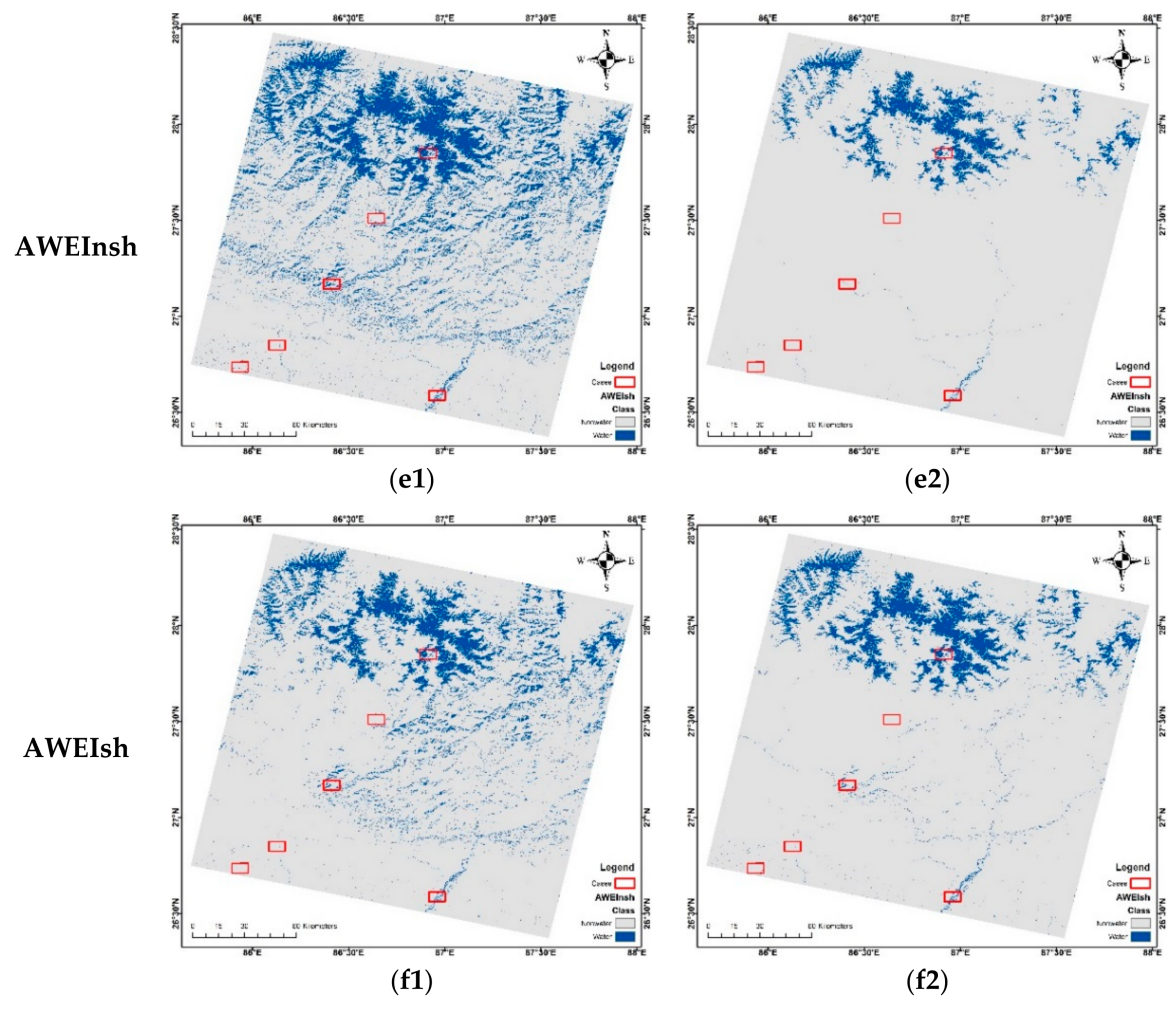
Surface water classification for different water indices with the standard threshold (**a1**–**f1**) and the optimum threshold selected from Table 4 (**a2**–**f2**).

Figure 4 shows all the surface water maps derived from both standard and optimum threshold methods. For each case, using the validation points (Figure 2), accuracy assessment measures as per Section 2.4 were derived. The results of the assessment are shown in Table 5. 

Based on the visual inspection, all the indices with the selected threshold seem to show different patterns. First, for the standard threshold, MNDWI performed the worst. Most of the other indices seem to perform well in flat and hilly regions, but not in the Himalayas with ice water and snow cover. NDVI (Figure 4a1) and NDWI (Figure 4b1) seem to extract mostly pure water pixels in the flat and hilly lands, whereas AWEInsh and AWEIsh seem to be able to extract mixed pixels as well, but at the cost of hilly shadows. Meanwhile, the optimum threshold seems to improve each of the water indices’ derivatives. Visually they are very clear and represent much of the water pixels, removing most classification errors for shadow and other non-water surfaces. NDVI (Figure 4a2) and NDWI (Figure 4b2) seem to show better and clear results again for pure water pixels in both flat and Himalaya regions, but left many mixed pixels for narrow and small water bodies. Both MNDWI and AWEI variables seem unable to reject snow cover in the optimum thresholding as well (Figure 4). In quantitative evaluation, the optimum threshold shows better accuracy in the extraction results. From Table 5, PA is higher for all indices with the standard threshold, which decreases with manual selection, but it is opposite for UA, OA, and kappa, which are increased. Both MNDWI and AWEInsh showed low performance in both threshold cases. NDVI and NDWI with optimum thresholds performed better with more than 87% OA and a kappa of 0.589 and 0.5966, respectively. However, PA seems to fall, whereas UA seems to increase for both drastically. Taking all the measures into consideration, AWEIsh seems to balance the performance well in the Landsat 8 scene of Nepal.

**Figure 5:**
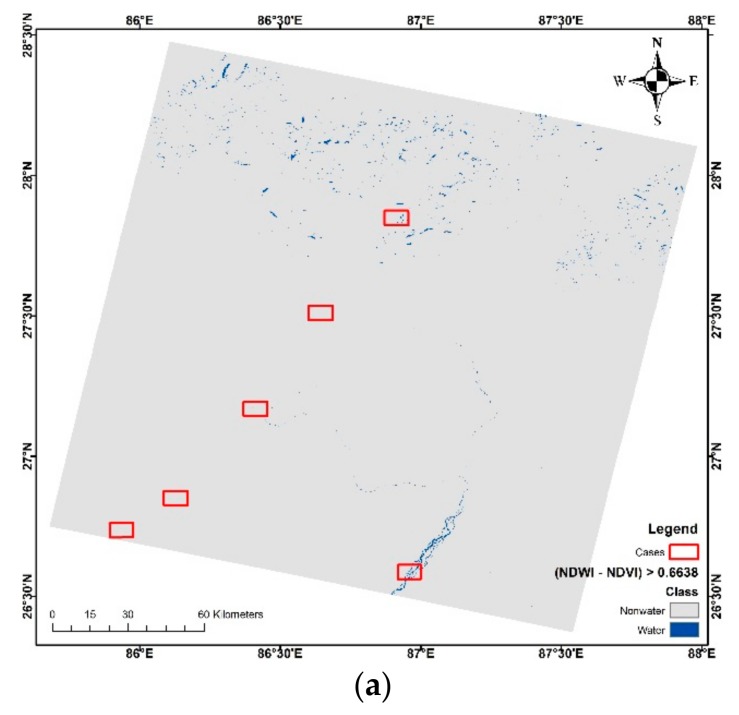

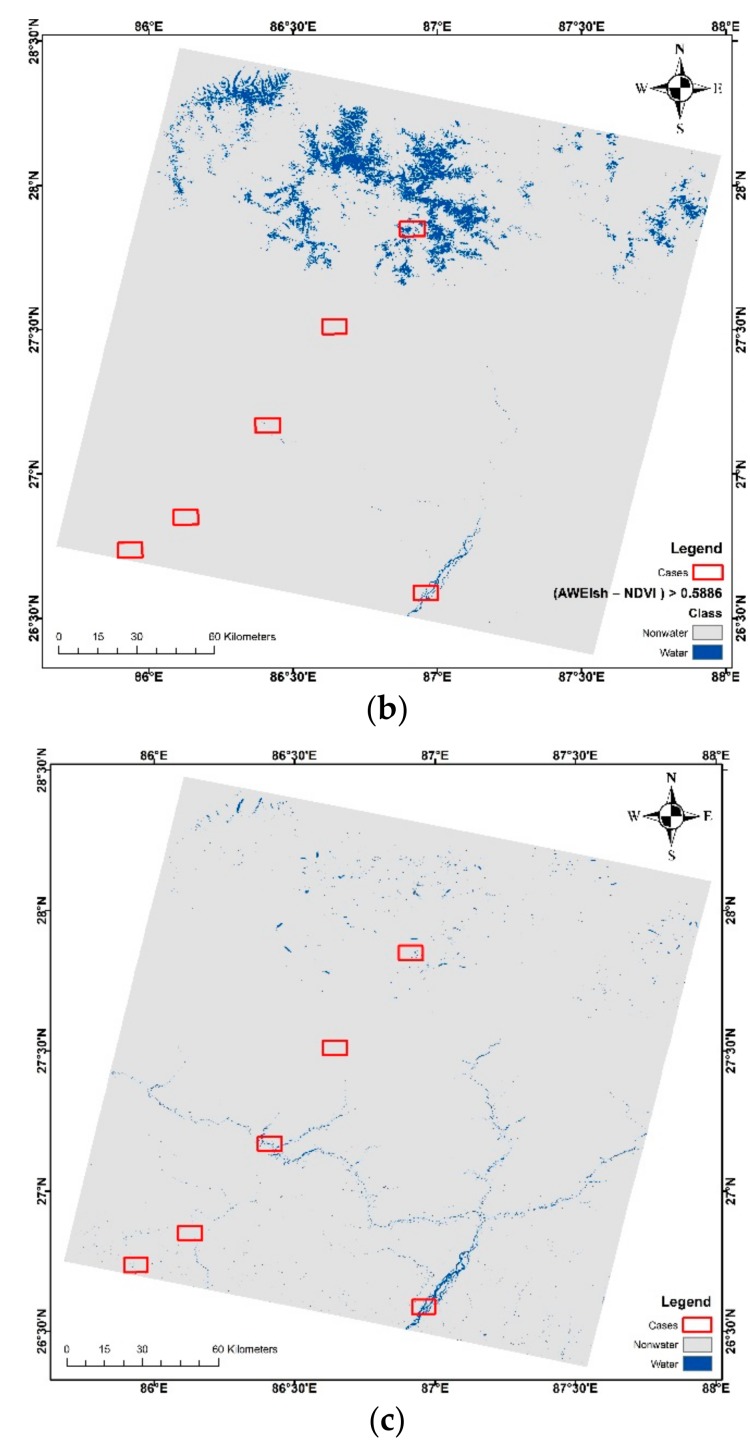
Surface water classification for proposed water indices with threshold selected from Table 5: (**a**) NDWmVI; (**b**) AWEIshmVI; and (**c**) Elev_NDWnVI.

Despite the increase in OA and kappa with balanced PA and UA in optimum thresholding, none of the indices showed OA above 90%. As most of the studies in the literature show OA to be higher than 90%, and even up to 99% [5,33,40,41], none of them were able to do so in the scene. This shows that such scenes require a combination of, or special conditions for, extraction. Taking this into consideration, we selected NDVI, NDWI, and AWEIsh indices which showed good performance in Table 5 for possible combination. The negative value of NDVI and positive values of NDWI and AWEInsh for water can be used to enhance the separability by difference as shown in Table 6. Additionally, considering the variation in elevation in the scene, we used SRTM DEM for creating segmentation of the test scene and used a combination of indices to extract surface water with better visual and quantitative accuracy. The trial and error method was performed for these cases to produce the highest OA and kappa using the validation points. The result of the surface water maps is shown in Figure 5 and their accuracy assessment is shown in Table 6.

Among the three proposed methods, the result from segmentation using elevation with NDWI and NDVI (Elev_NDWnVI) shows the highest OA of 0.9683, followed by the NDWI—NDVI (NDWmVI) and AWEIsh—NDVI (AWEIshmVI). The kappa also seems to be very high for Elev_NDWnVI (0.8979) while NDWmVI is 0.6079 and AWEIshmVI is only 0.4265. Despite the higher OA, AWEIshmVI shows poor PA and kappa, both less than 0.5. In terms of visual interpretation, the Elev_NDWnVI result is also cleaner and shows surface waters in the whole scene well, while the rest contain errors in either the Himalayas or flat lands, inheriting their composite indices’ features. The NDWmVI-derived map shows more non-water pixels compared to the others. While it shows snow water and large rivers, the mid-hilly narrow and turbid waters are not well represented. In contrast, the AWEIshmVI map misclassifies both water and non-water bodies in the Himalayan areas with snow cover and mid-hilly regions’ rivers. Additionally, its performance is less than the NDVI and NDWI optimum thresholds.

**Figure 6:**
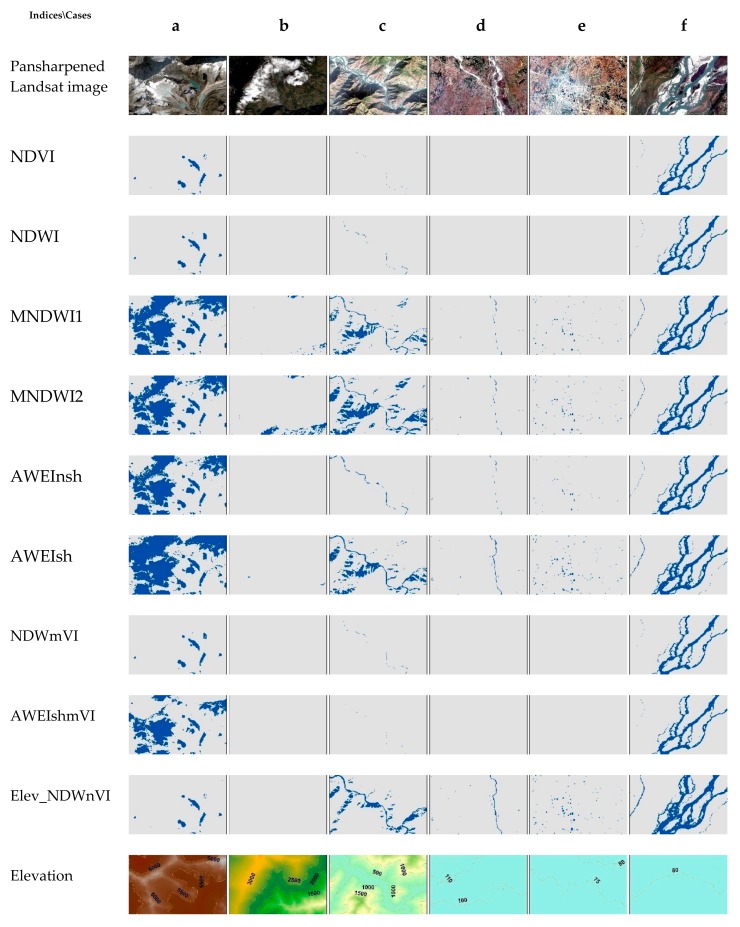
Comparison of special cases of surface water (**a**–**f**) in the test scene (Figure 2) for different water indices with optimum thresholds (Table 5) and the proposed methods (Table 6).

Overall, the test scene selected from Nepal shows better results when segmented with elevation, as well as somewhat to the combination compared to the optimum and standard thresholds of the water indices. To check how well all these methods have done in the scene, we selected six different types of cases as shown in Figure 2 and described in Section 2.2. We will be seeing them one by one from case a to f. Visual comparisons of these cases are shown in Figure 6.

Case a represents the ice water in the snow-covered Himalayas. In this case NDVI, NDWI, and NDWmVI were better able to detect snow water against the shadow and the snow while the other failed and misclassified all as water. This also seems to be a good reason to select either, or both, for the water detection in the higher Himalayas. In case b, except MNDWI2, the rest of the indices seem to be able to discard cloud and shadows. The performance of surface water detection for narrow river in hilly regions with shadows is shown in case c, in which all indices were able to detect pure water pixels. However, in the case of mixed water pixels, NDWI and NDWmVI were somewhat successful but not as much as rest of others. Both MNDWI (1 and 2), as well as AWEIsh, wrongly classified shadows as water. AWEInsh detected both pure and mixed pixels. While segmenting the test scene with elevation below 665 m, the NDWI threshold was lowered to −0.05 from the optimum of 0.3877 and resulted in wrong classification of shadow as water. Similarly, with elevation less than 665 m, neither NDVI, NDWI, NDWmVI, nor AWEIshmVI were able to detect the water in narrow or shallow rivers due to mixed pixels (case d). In such a case, Elev_NDWnVI and AWEIsh gave good detection results. However, AWEInsh, including both MNDWI, detected only pure pixels. Case e shows an urban area with many artificial ponds with turbid waters. These ponds were not well detected by the optimum threshold of NDWI, NDVI, and the combined indices. As MNDWI and AWEI cases were derived as alternative to NDWI to solve the problems in urban areas [15,17], both methods detected them well. In this case, the lowered threshold of NDWI for elevation less than 665 m also showed good detection ability. Finally, case f, represents the wide and shallow branches braided with sand of Koshi River on flat land. The central deep river surface caused by Koshi Barrage was well represented by all indices. Then again, the issue was same for the branches of narrow and shallow rivers, which resembles case d.

As stated in the Introduction, most of the studies focus on scenes with large water bodies or a subset with a higher percentage of water; the case scene was very challenging as it has only 0.67% surface water. Moreover, the challenging issues were the large difference in elevation, surface water body types, and the presence of various form of environmental noise in the scene. In principle, water index methods are simple and show high accuracy while extracting the surface water with standard or optimum thresholds. However, after evaluation in this study, it is evident that it is not always the case. These are not practical when there are different water types and environmental noises. This is more evident by the higher PA for all indices with standard thresholds. At the same time, non-water surfaces were also misclassified resulting in UA and kappa less than 50%. It signifies the classification of non-water surfaces were purely random chance compared to water surfaces. This could also be possible due to the classification of the shadow surface as water. Therefore, it seems important to consider including a shadow detection method to resolve it.

To reduce such misclassification, a proper threshold needs to be found that represents the actual surface water. Usually for small areas, an expert’s view and close matching with high-resolution imagery gives a better idea on the optimum threshold. However, the second challenge in this study was in that optimum threshold selection. The coverage was so large, and water coverage was sparse, with a great deal of environmental noise. For that we used trial and error with our validation dataset as a reference meaning the threshold value that will give the highest overall accuracy and kappa will be selected. For the selected optimum threshold values, NDVI, NDWI, and their derivative, i.e., NDWmVI, were able to detect only pure pixels. The derived indices from MNDWI and AWEI were able to detect mixed pixels along with environmental noise, resulting in lower UA.

In the plot for MNDWI1 (Figure 3b), we can see that both OA and kappa increase until the optimum threshold and decrease in parallel after the threshold. Though it should be a smooth curve, the case MNDWI2 shows a drastic jump in kappa for the 0.5 threshold, which raises the issue of the selection of validation points. Another reason showing the limitation of the validation points is that none of the optimum thresholds cross 90% OA with a kappa higher than 0.7. Furthermore, the best result from this study with 96% OA, i.e., Elev_NDWnVI, also misclassifies hill shadows as water (Figure 6, case c).

Another challenge that has not been dealt with in this study and could be possible in surface water detection is the effect of weather/atmosphere. The geographic and land cover variation highly relates to the weather variation. The scene can have clear, hazy, or cloudy regions depending on elevations and wind patterns. Hence, it is also necessary to carefully note the atmospheric effects and pre-process them to reduce the effect as much as possible.

Among the three proposed methods, AWEIshmVI did not perform well, NDWmVI was somewhat improved compared to the individual NDVI and NDWI, but Elev_NDWnVI showed a drastic improvement. This clearly supports our initial assumption that elevation plays an important role in selecting the water index method and their optimum values. Even though the accuracy was high compared to the others, it was susceptible to shadows (Figure 6, case c). The issue of shadows can be resolved by generating and applying a shadow mask from the elevation data. However, based on this study’s findings, surface water feature extraction using index methods can be more refined with smaller classes of the elevation and can be further applied for any other characteristics that can segment a scene.

## 4. Conclusions

For a Landsat 8 scene, there could be multiple types of surface water coupled with many sources of environmental noise. For a country like Nepal, the scene shows further diversity with large elevation difference in a short horizontal distance. This study evaluated the performance of the most widely used water indices: NDVI, NDWI, MNDWI, and AWEI in a Landsat 8 scene of Nepal. The optimum threshold was identified with validation points and compared with the standard threshold for each method. Furthermore, we proposed a combination of NDWI and AWEIsh with NDVI, as well as segmenting with elevation for possible surface water detection. The comparison was performed visually for different cases and quantitively using measures from a confusion matrix. The results of the study can be summarized as follow:

(a) The standard threshold of all the water indices was able to extract most of the surface water pixels, i.e., high PA, but with many misclassified non-water pixels, i.e., low UA. Based on visual and quantitative evaluations, the standard threshold is not useful in deriving a surface water map in scene with diverse characteristics.
(b) The optimum threshold improves the detection ability in most of the cases with higher OA, but lower kappa. With an optimum threshold, NDVI and NDWI were good at detecting pure pixels and rejecting the rest, whereas MNDWI and AWEI were able to detect mixed pixels of small ponds and rivers, but unable to reject snow cover and shadow in the Himalayas.
(c) The combined approach for NDWI and AWEIsh with NDVI did not improve the detection ability in the scene.
(d) Segmentation of the scene with elevation showed that NDVI and NDWI can be used for accurate surface water detection for different elevation range and water types. The proposed elevation above and below 665 m used NDVI and NDWI for the surface water detection, respectively. The OA for the proposed method was 0.9638 and the kappa was 0.8979. However, it was susceptible to shadows.

This study shows that the surface water detection can be enhanced by segmentation with elevation which can be extended to other characteristics that can segment a scene. Additionally, it is recommended to use a smaller interval with sufficient validation samples representing all types for adequate performance and shadow removal techniques. These findings and recommendations will be used to investigate the applicability of the proposed method in multiple scenes and compared with state-of-art machine learning methods.

## Figures and Tables

**Table 1:** Specifications of the Landsat 8 OLI senor image [4,35].

Satellite/Sensor	Band	Wavelength (µm)	Name	Resolution (m)
Landsat 8/OLI	1	0.435–0.451	Coastal Aerosol (CA)	30
	2	0.452–0.512	Blue	
	3	0.533–0.590	Green	
	4	0.636–0.673	Red	
	5	0.851–0.879	Near Infrared (NIR)	
	6	1.566–1.651	Shortwave NIR 1 (SWIR1)	
	7	2.107–2.294	Shortwave NIR 2 (SWIR2)	
	9	1.363–1.384	Panchromatic	15

**Table 2:** Multiband indices used for water feature extraction.

Multiband Index	Equation	Water Value	Reference
Normalized Difference Vegetation Index	NDVI = (NIR − Red)/(NIR + Red)	Negative	[38]
Normalized Difference Water Index	NDWI = (Green − NIR)/(Green + NIR)	Positive	[19]
Modified Normalized Difference Water Index	MNDWI1 = (Green − SWIR1)/(Green + SWIR1) MNDWI2 = (Green − SWIR2)/(Green + SWIR2)	Positive	[17]
Automated Water Extraction Index	AWEIsh = Blue + 2.5 × Green − 1.5 × (NIR + SWIR1) − 0.25 × SWIR2 AWEInsh = 4 × (Green − SWIR1) − (0.25 × NIR + 2.75 × SWIR1)	Positive	[15]

**Table 3:** A confusion matrix.

		Reference Data	
		Water	Non-Water
**Classified data**	**Water**	TP	FP
	**Non-water**	FN	TN

**Table 4:** Selected optimum threshold values for different water indices in the test scene.

Multiband Index	NDVI	NDWI	MNDWI1	MNDWI2	AWEInsh	AWEIsh
Optimum thresholds	−0.2955	0.3877	0.35	0.5	0.1897	0.1112

**Table 5:** Accuracy assessment for standard and optimum threshold values for different water indices based on the validation dataset in the test scene.

Index	Standard Threshold				Optimum Threshold			
	PA	UA	OA	Kappa	PA	UA	OA	Kappa
**NDVI**	0.8495	0.4907	0.76	0.4641	0.5	0.949	0.8775	0.589
**NDWI**	0.9301	0.5	0.7675	0.4988	0.5323	0.8919	0.8762	0.5966
**MNDWI1**	0.9785	0.4539	0.7212	0.4432	0.7742	0.4865	0.7575	0.4366
**MNDWI2**	0.9946	0.3439	0.5575	0.2529	0.8763	0.4697	0.7412	0.443
**AWEInsh**	0.8495	0.4788	0.75	0.4484	0.6183	0.5134	0.775	0.4115
**AWEIsh**	0.9839	0.4598	0.7275	0.4535	0.9409	0.5287	0.7912	0.5401

**Table 6:** Accuracy assessment based on the validation dataset for the proposed combinations of indices and their threshold values for water extraction in the scene.

S. No.	Given Abb.	Segmentation	Index	Threshold	PA	UA	OA	Kappa
1	NDWmVI	-	NDWI—NDVI	0.6638	0.5376	0.9091	0.8800	0.6079
2	AWEIshmVI	-	AWEIsh—NDVI	0.5886	0.4946	0.6093	0.8088	0.4265
3	Elev_NDWnVI	Elevation > 665 m	NDVI	−0.295	0.9140	0.929	0.9638	0.8979
		Elevation < 665 m	NDWI	−0.05				
